# Nonionic Microemulsions as Solubilizers of Hydrophobic Drugs: Solubilization of Paclitaxel

**DOI:** 10.3390/ma9090761

**Published:** 2016-09-07

**Authors:** Jen-Ting Lo, Tzer-Min Lee, Bing-Hung Chen

**Affiliations:** 1 Department of Chemical Engineering, National Cheng Kung University, Tainan 70101, Taiwan; luegg277@yahoo.com.tw; 2 Institute of Oral Medicine, National Cheng Kung University, Tainan 70101, Taiwan; tmlee@mail.ncku.edu.tw

**Keywords:** paclitaxel, microemulsion, solubilizer, nonionic-surfactant, oil phase, co-surfactant

## Abstract

The strategy using nonionic microemulsion as a solubilizer for hydrophobic drugs was studied and is demonstrated in this work. The aqueous phase behaviors of mixed nonionic surfactants with various oils at 37 °C are firstly constructed to give the optimal formulations of nonionic microemulsions with applications in the enhanced solubilization of the model hydrophobic drug, paclitaxel, at 37 °C. Briefly, the suitable oil phase with paclitaxel significantly dissolved is microemulsified with appropriate surfactants. Surfactants utilized include Tween 80, Cremophor EL, and polyethylene glycol (4.3) cocoyl ether, while various kinds of edible oils and fatty esters are used as the oil phase. On average, the apparent solubility of paclitaxel is increased to ca. 70–100 ppm in the prepared microemulsions at 37 °C using tributyrin or ethyl caproate as the oil phases. The sizes of the microemulsions attained are mostly from ca. 60 nm to ca. 200 nm. The cytotoxicity of the microemulsion formulations is assessed with the cellular viability of 3T3 cells. In general, the cell viability is above 55% after 24 h of cultivation in media containing these microemulsion formulations diluted to a concentration of total surfactants equal to 50 ppm and 200 ppm.

## 1. Introduction

Nowadays, the development of new drugs often encounters great difficulty during tests of drug efficacy and cytotoxicity owing to the low aqueous solubility of the newly discovered entities of drugs [1,2,3]. As a result, various solubilizers with relatively low toxicity are developed to increase the solubility of these poorly soluble drugs in formulations for preclinical studies [1,3,4,5,6,7,8]. Most solubilizers are prepared with the use of surfactants. Specifically, the micellar solubilization as well as the encapsulation by microemulsion/liposome account for most mechanisms of action in surfactant-based solubilizers hydrophobic drugs. Notably, the micellar solubilization was known earlier to formulation scientists and work well in small molecular drugs, while microemulsion/liposomes are often applied to the encapsulation of larger drug molecules [1,3,4,5,6,7,9,10]. For example, microemulsions have been successfully applied to clinical parenteral formulations of paclitaxel (MW = 853.9 g/mol), calcitriol (MW = 416.64 g/mol), cyclosporine A (MW = 1202.63 g/mol), etc. [5]. Furthermore, microemulsion has recently drawn considerable interests in drug delivery systems and as a drug solubilizer, owing to advantages such as an easy preparation [11] and a good solubilization power of hydrophobic drugs [1,3,4].

Microemulsions are thermodynamically stable and have excellent solvent power [11]. Furthermore, the droplet sizes of nonionic microemulsions are generally less than 200 nm, which is thought to be less susceptible to the uptake and clearance by the reticuloendothelial system (RES) [12]. Hence, microemulsion is commonly regarded as an ideal candidate for intravenous administration of hydrophobic drugs, e.g., paclitaxel [11,13]. However, successful preparation of microemulsion really depends on various factors and often requires proper selection of surfactants, co-surfactants, and oil phases in which hydrophobic drugs are contained. That is, the preparation scheme of a microemulsion could vary based upon different conditions of storage and application, even with the same solubilizates to be enclosed. Hence, intensive experimental work is often carried out to obtain the optimal formation conditions of microemulsions [11].

In this work, one aim of ours is to devise and demonstrate the general procedure and preparation method of nonionic microemulsions that can be applied as solubilizers of hydrophobic drugs. The model hydrophobic drug selected for this study is paclitaxel, as it is known as an effective antineoplastic agent against a wide range of cancers such as ovarian carcinoma, breast carcinoma, non-small cell lung cancer, gastric cancer, etc. [14,15,16,17]. However, its efficacy is often limited by its extremely low aqueous solubility (<0.3 ppm) [18]. As such, there have been many approaches for increasing the apparent aqueous solubility of paclitaxel, including hydrotropic solubilization [13], microemulsification [4,5,10], polymeric nanoparticles [19], etc. Furthermore, paclitaxel has been extensively studied since the 1990s and, thus, its molecular property is well known and found in the literature [4,13].

In brief, this report can be outlined as the following three parts. First, preparation of nonionic microemulsion solubilizers starts from the selection of proper oil phases in possession of high solubility of paclitaxel. Subsequently, appropriate phase diagrams of nonionic surfactants and oil phases at 37 °C are constructed to give the optimal conditions for microemulsion preparation. Finally, the cytotoxicity of the obtained microemulsion formulation is tested to validate the preparation of surfactant-based solubilizers for hydrophobic drugs.

## 2. Results

### 2.1. Selection of Oil Phase

Selection of an appropriate oil phase was mainly based on the combined contribution from the higher solubility of paclitaxel in these oils and the possibly larger solubilization capacity of oils by surfactant solutions. Table 1 summarizes the solubility of paclitaxel in oils and fatty esters at 37 °C.

In various ethyl fatty esters, the solubility of paclitaxel increases with decreasing aliphatic chain length of fatty acids, for instance, from 0.74 mg per mL of ethyl myristate to 14.57 mg per mL of ethyl caproate. Similarly, this trend was again observed for triglycerides. A high solubility of paclitaxel near 28 mg/mL (i.e., 26.92 mg/g·oil) in tributyrin was garnered in this work, slightly higher than 20.5 mg/mL reported by Simamora et al. [20], and 11.8 mg/g·oil at 25 °C [4]. It is of note to mention that the solubility of paclitaxel apparently increases almost 2-fold from 11.8 mg/g·oil to 26.92 mg/g·oil with an increasing temperature from 25 °C to 37 °C. 

Conversely, edible oils are mostly triglycerides and consist largely of the unsaturated fatty acids, such as oleic acid (C_18:1_) and linoleic acid (C_18:2_). They do not possess a good solubilization capacity of paclitaxel, as shown in Table 1 and in our previous work [4]. For instance, the solubilities of paclitaxel in these edible oils were found to be near 0.3–0.5 mg/g·oil, even less than the solubility of paclitaxel in ethyl myristate.

As mentioned previously, one aim of this work is to formulate a nonionic oil-in-water (O/W) microemulsion system that could solubilize paclitaxel as much as possible. Therefore, a rule of thumb is to start the preparation procedure from using the aqueous surfactant solutions having a larger solubilization capacity of the oil phases in which a greater solubility of paclitaxel is observed. Consequently, both tributyrin and ethyl caproate were chosen as oil phases for the subsequent experiments in the preparation of microemulsions. Notably, tributyrin is naturally present in butter and commonly used as a food additive. Ethyl caproate is naturally found in pineapple and a common food-grade fragrance oil that emits the fruity odor. Moreover, the exploration of proper aqueous surfactant systems having larger solubilization capacities of tributyrin and ethyl caproate were attempted with an aid of phase diagrams. The procedures are described in the next section.

### 2.2. Characteristics of Prepared Microemulsions

**Figure 1:**
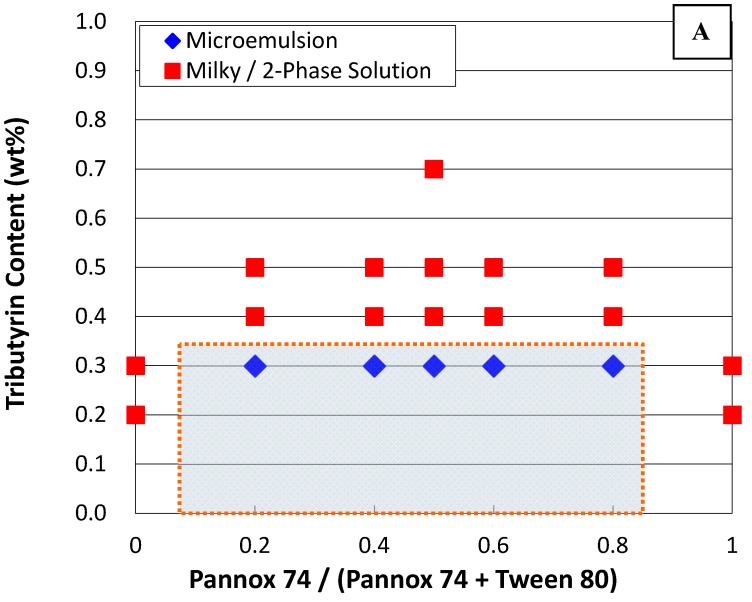

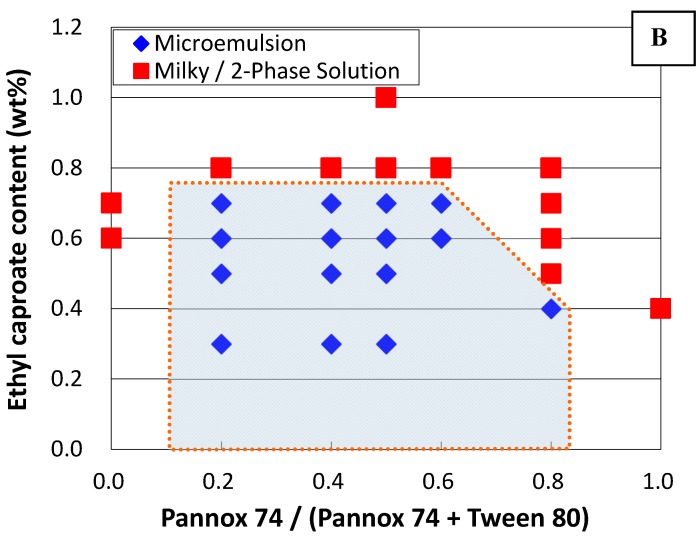
Phase diagram of a system containing tributyrin (**A**), ethyl caproate (**B**), and mixed surfactants of Pannox 74 and Tween 80, as well as 1 wt % glycerol as a co-surfactant. The overall surfactant concentration is kept constant at 2 wt %.

**Figure 2:**
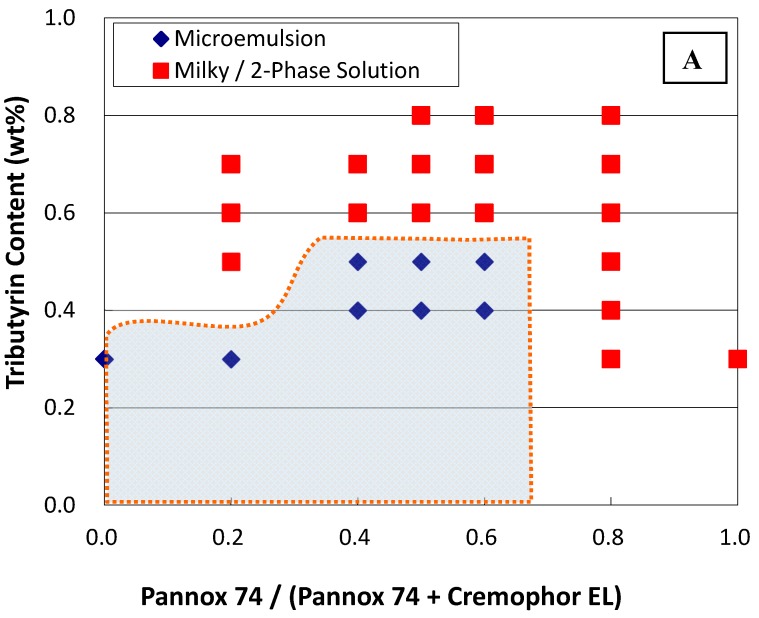

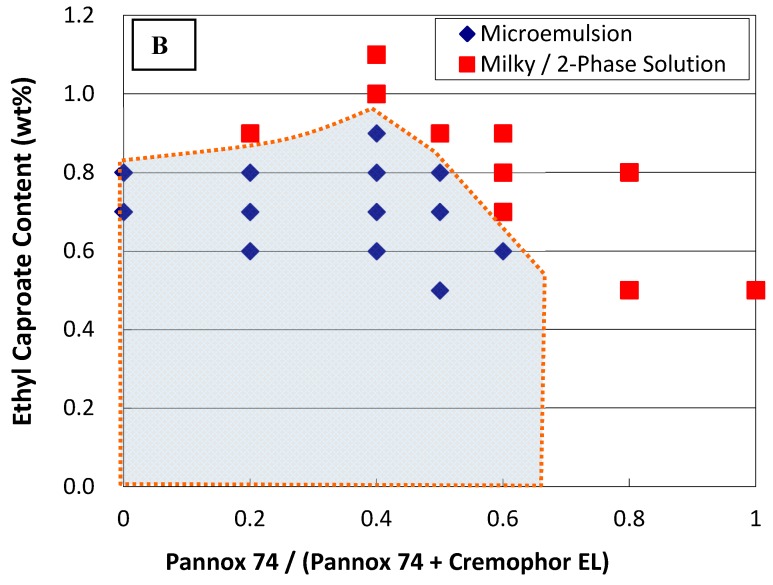
Phase diagram of a system containing tributyrin (**A**), ethyl caproate (**B**), and mixed surfactants of Pannox 74 and Cremophor EL, as well as 1 wt % glycerol as a co-surfactant. The overall surfactant concentration is kept constant at 2 wt %.

Phase diagrams were established by examining the phase behaviors of various composition points. Each composition point contains a specific content of mixed surfactants and oil. The phase behavior of the composition point was determined from the observed phase stability and the size measurement of the aggregates by the dynamic light scattering (DLS) technique. The phase boundary was drawn in between two adjacent composition points showing distinct phase behaviors, namely one phase point showing microemulsion and the other adjacent phase point exhibiting unstable agglomeration (milky solution)/two-phase regions. In general, the sample vials were left still at 37 °C for a period up to a week and observed visually. Vials showing single isotropic phases are deemed as microemulsions. However, those systems showing one-phase bluish-white dispersions were further examined with DLS for phase determination. In a word, the shadow area on Figure 1 and Figure 2 are the projected microemulsion regions.

Figure 1 and Figure 2 show the phase diagrams at 37 °C resulting from mixed surfactants and tributyrin/ethyl caproate as oil phases. The concentration of mixed surfactant in this work was maintained constant at 2 wt %, while glycerin in 1 wt % was added as co-surfactant to stabilize the microemulsions. Notably, several surfactants including Span 20, Span 80, Pluronics, and ethoxylated oleic acid (with the hydrophile–lipophile balance (HLB) value at 11.7) were tested as well, but no stable microemulsions with tributyrin/ethyl caproate as oil phases could be formed.

In general, the stable phase regions of microemulsions prepared with the use of ethyl caproate extends to a higher oil concentration than that with the use of tributyrin. Likewise, with mixed surfactants of Pannox 74 (HLB = 9.8) and Tween 80 (HLB = 15), the stable phase regions were found with a mass ratio of Pannox 74 in surfactant mixtures from 0.2 to 0.8 using both tributyrin and ethyl caproate as oil phases. Replacing Tween 80 with Cremophor EL (also known as Kolliphor EL, HLB = 13.5), the stable microemulsion phases exists with a mass ratio of Pannox 74 in surfactant mixtures from 0 to 0.6. That is, only with Cremophor EL itself, can it still form stable microemulsions with tributyrin up to 0.3 wt % and ethyl caproate up to 0.8 wt %. Though Cremophor EL has been approved as the solubilizing agent of paclitaxel in Taxol for intravenous administration, the side effects like allergic reactions induced by Cremophor EL are still of grave concern [4].

Table 2 tabulates the hydrodynamic diameters of aggregates in selected microemulsion formulations measured by DLS techniques. These phase points for DLS analyses are proximal to phase boundary but still located in the microemulsion phase. In general, with Pannox 74 and Tween 80, the colloid sizes of the formulation in the microemulsion regions were approximately 60–150 nm at one day after preparation, and 60–260 nm at one week after preparation. With the substitution of Cremophor EL for Tween 80, the aggregate sizes of the prepared microemulsions ranged roughly from 93.4 nm to 270 nm and from 82.4 nm to ca. 300 nm after one day and one week from preparation, respectively. The DLS measurements certainly confirmed the existence of microemulsions in these formulations [11]. Though the aggregate sizes in some formulations could reach almost 270 nm, these emulsion formulations seemed quite stable and did not phase-separate. Notably, stable nonionic microemulsions could have aggregates as large as near 300 nm [4]. The growth of the colloidal dispersion in size, shown in Table 2, could be attributable to the fact that they have already been close enough to the two-phase region. In this work, the prepared microemulsions were stable for at least over a week after preparation.

### 2.3. Solubilization of Paclitaxel by Emulsions Formulations

As aforementioned, the aqueous solubility of paclitaxel is less than 0.3 ppm [18]. In contrast, the enhanced solubilization of paclitaxel by these microemulsion formulations were observed and shown in Table 3. The apparent solubilities of paclitaxel were measured with high-performance liquid chromatography (HPLC) and obtained as a value ranging from ca. 52 ppm to ca. 92 ppm. In general, the mixed surfactant system of Pannox 74 and Tween 80 with 0.7 wt % ethyl caproate as well as that of Pannox 74 and Cremophor EL with 0.4 wt % tributyrin gave the relatively high enhancement in the solubilization of paclitaxel, near 90 ppm.

The solubilization efficiency of paclitaxel (PTX) by the microemulsion was defined as the ratio of the measured PTX content to the estimated PTX solubility. The estimated solubility of paclitaxel was calculated based on the assumption that all paclitaxel dissolved in the oils would be completely solubilized and encapsulated within the oil phases of the microemulsions. That is, the estimated solubility of paclitaxel is equal to the product of the empirical solubility of paclitaxel in oils and the maximum solubility of such oils in microemulsions. As a result, the solubilization efficiency of paclitaxel was found in between 53% and about 90%. That is, a portion of paclitaxel that was initially dissolved in oils was lost during microemulsification. It had likely leaked out of the oil phases and precipitated into the surfactant solutions.

### 2.4. Cytotoxic Assessment of Selected Microemulsion Formulations

Four representative microemulsion formulations (Table 4) were selected for cytotoxic assessments. The concentrations of total surfactants were further diluted to 50 ppm and 200 ppm with culture media, along with other constituents in microemulsions, e.g., glycerol as well as ratio of constitutive surfactants, diluted in the same proportions. It has to be mentioned that no paclitaxel is contained in these four microemulsions for cytotoxic assessments to distinguish cytotoxicity of the formulations from that of paclitaxel. In brief, these four microemulsion formulations were selected not only because of satisfactory solubilization of paclitaxel but also to provide the cross examination on the influences by two mixed surfactant systems and two different oils. Hence, such an experimental design provides us not only cytotoxicity assessment of microemulsions, but also with discernment on the possible origin of cytotoxicity, if existing, resulting from the use of specific oil phases and different sets of mixed surfactants used in the solubilizers.

**Figure 3:**
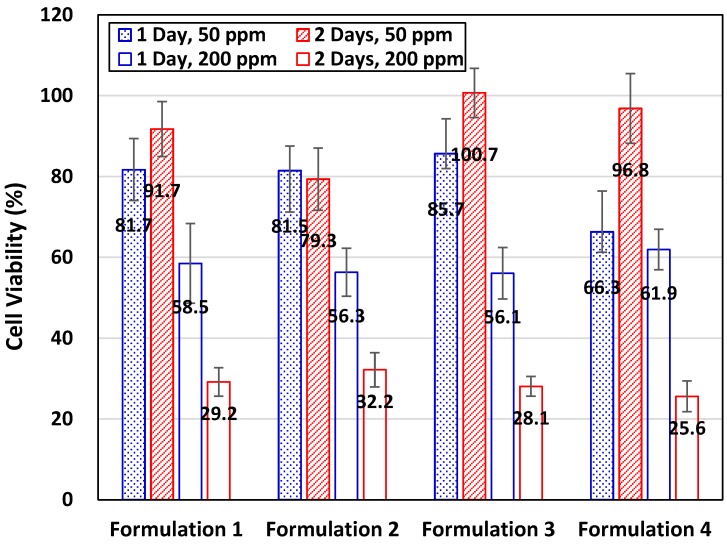
Viability of fibroblast 3T3 cells in selected microemulsion formulations, shown in Table 4, after 24 h and 48 h incubation. The emulsion formulations without paclitaxel loaded were added into inocula with a final concentration of total surfactants adjusted to 50 ppm or 200 ppm.

Figure 3 shows the relative viability of fibroblast 3T3 cells incubated in culture media with these formulations added to an overall surfactant concentration at 50 ppm and 200 ppm, respectively. Microemulsion solubilizers were introduced to inocula at 24 h after the initial seeding of 3T3 cells to culture wells, and the time of the 3T3 cell culturation starts to be counted. The relative cell viability in each specific formulation shown in Figure 3 was the ratio of the cell number with surfactants diluted with culture media to that found in the control sample without any surfactant under the same culture condition. The average numbers of 3T3 cells per well incubated under the control conditions for one and two days are ca. 14,500 and 34,500, respectively.

With the total surfactants diluted to 50 ppm in the culture media, the growth of 3T3 cells was suppressed in all four formulations after the first day of incubation but recovered soon on the second day of incubation, except in Formulation 2. However, increasing the concentration of total surfactants to 200 ppm in culture media, 3T3 cells did not grow as well as those in the control samples. The relative viability of 3T3 cells incubated in all four formulations with a concentration of total surfactants diluted to 200 ppm was reduced to about 56%–62% and further to ca. 26%–32% after incubation for one and two days, respectively (Figure 3).

Interestingly, the average numbers of survival cells per well were in the range from 8100 to 8900 for all formulations after one day’s incubation, while the average number of 3T3 cells in control samples was increased to about 14,500. With two day’s incubation, 3T3 cells grew very slowly to bring the average cell number to about 9000–11,000 per well in all formulations, compared to an average of 34,500 cells per well in the control samples. In view of survival cell numbers, these formulations retained the propagation of 3T3 cells, but was not so detrimental to eliminate all 3T3 cells in test wells.

In general, with more surfactants added to the culture media, less cell viabilities could be observed in this work. Even though the surfactant used are generally less toxic, a high concentration of surfactants may still hinder the in vitro growth of 3T3 cells. Surfactants are amphiphilic molecules that contain both hydrophilic and lipophilic groups. They tend to form aggregates with lipids, such as micelles and liposomes. Once contacting living cells, surfactants with proper structure configuration could be inserted to the phospholipid bilayers, mainly driven by the hydrophobic interactions between surfactants and lipids. With enough amounts, surfactant could permeate and lyse cell membranes, and even solubilize the embedded membrane proteins. As such, surfactants could disrupt the biological functions of cytoplasmic membranes and cause the death of cells [9].

## 3. Discussion

The oil phases screened in this work show a PTX solubility of up to 26.92 mg/g·oil, comparable to PTX solubility in various organic hydrotropic agents reported by Lee et al. [13]. However, the direct use of organic solvents could be detrimental to cell growth and, thus, jeopardize the tests of toxicity and potency of new drug entities. Furthermore, hydrophobic drugs initially dissolved in these hydrotropic solvents could precipitate out when diluted with water, saline solution, and aqueous diluents. For example, paclitaxel in Taxol has been well known to likely precipitate in the infusion line and bag during intravenous administration, if great attention has not been paid [4,18,20]. In this work, the use of nonionic microemulsion solubilizers has achieved the solubilization efficiency of paclitaxel near 60%–90%. That is, most of paclitaxel still remains in the aqueous solutions, available to the tests of toxicity and efficacy of drugs.

The preparation procedure of microemulsions really depends on various factors [11]. An empirical equation proposed for polyoxyethylene (POE) nonionic surfactant-based microemulsions was given as Equation (8.12) in the textbook authored by Rosen and Kunjappu [11]. This practical equation is useful to provide the rule of thumb in microemulsion preparation. However, the equation itself has also revealed the complexity in the preparation of microemulsions. In brief, the proper conditions to obtain the microemulsion solubilizers really rely on adequate choices of oils, surfactants, and co-surfactants with the procedures underlined in this work. Alternatively, the preparation principles of the microemulsions underlined in this this work could be applicable to prepare the solubilizers of the hydrophobic drugs.

In general, the size of microemulsion is considered as that smaller than 100 nm, but larger than micelles. However, from our previous work, we have obtained nonionic surfactant-based microemulsions with sizes up to 250 nm, measured by DLS [4]. Indeed, ionic-surfactant-based microemulsions could be much smaller, e.g., ca. 20 nm for microemulsions made of sodium dodecyl sulfate (SDS). In this work, the primary consideration on the use of edible nonionic surfactants in the preparation of solubilizers is reflected with a hope for a further application not only in vitro/ex vivo but also in vivo to humans. Another concern in selection of surfactants is reflected with the toxicity of surfactants. Ionic surfactants are generally regarded as more cytotoxic to cell cultures and, therefore, used extensively in personal care products and household cleaning products with an emphasis on bactericidal functions [4]. In general, nonionic surfactants prevails, hence, over ionic surfactants in drug delivery systems.

## 4. Materials and Methods

### 4.1. Materials

The model hydrophobic drug, paclitaxel, was purchased from Scino Pharm Ltd. (Tainan, Taiwan). Edible oils were purchased from local supermarkets. Ethyl esters were obtained from various suppliers: ethyl caproate and ethyl myristate from Alfa Aesar (Heysham, Lancashire, UK), ethyl caprate from Fluka (Buchs, Switzerland), as well as ethyl caprylate and ethyl laurate from Acros (Geel, Belgium). Tributyrin, tricaprylin and triolein, glycerol, Tween 80 surfactant (HLB = 15.0), thiazolyl blue tetrazolium bromide (MTT) and Cremophor EL emulsifier (HLB = 13.5), also known as Kolliphor EL, were acquired from Sigma-Aldrich (St. Louis, MO, USA). The nonionic surfactant Pannox 74 emulsifier (Polyethylene glycol (4.3) cocoyl ether, HLB = 9.8) was given by Pan Asia Chemical Corporation (Taipei, Taiwan) as a gift. Mouse fibroblast cell line (NIH/3T3) was purchased from the American Type Culture Collection (ATCC CRL-1658, Manassas, VA, USA). Dulbecco’s Modified Eagle Medium (DMEM) of low glucose and fetal bovine serum (FBS) were acquired from Gibco^®^ of Invitrogen Corporation (Carlsbad, CA, USA).

Deionized water from a Milli-Q ultra-purification system (EMD Millipore, Billerica, MA, USA) having resistivity greater than 18.2 MΩ·cm was used in sample preparation. All chemicals were of reagent grade and used as received without further purification.

### 4.2. Experimental Procedures

One objective of this work is to develop proper oil-in-water (O/W) microemulsion formulations that can solubilize and encapsulate as much hydrophobic drug as possible. Therefore, the experimental layout of this work can be briefly divided into three phases described as follows: (1) screening of oil phases to have a higher solubility of model drugs; (2) phase behavior of microemulsions composed of biocompatible surfactants, selected oil phases and the model drugs; and (3) performance and characterization of drug-contained microemulsions. These microemulsions should be stable enough both chemically and physically. Moreover, the cytotoxic assay of the formulations having a higher solubilization capacity of paclitaxel would be examined. All experiments in this work were conducted at 37 °C.

#### 4.2.1. Screening of Oil Phases

Paclitaxel is relatively hydrophobic and has a low aqueous solubility of less than 0.3 ppm [4]. Therefore, microemulsions and nanoemulsions with oil phases having good solubility of paclitaxel were formulated to enhance the apparent solubility of paclitaxel. The solubility of paclitaxel in various kinds of edible oils, ethyl esters, and triglycerides were investigated. Excess amount of paclitaxel was added into oil phases and mixed by a vortex mixer for few minutes and, subsequently, on an end-to-end rotary mixer (Model LD-76, Labinco B.V., Breda, The Netherlands) until oils were completely saturated with paclitaxel. These paclitaxel-containing oils were, subsequently, centrifuged at 6000 rpm for 30 min (Model Z 300K, Hermle Labortechnik GmbH, Wehingen, Germany). The supernatant was withdrawn, filtered with 0.45 μm PTFE syringe filters to remove any chunks of undissolved paclitaxel, and diluted appropriately with the mobile phase used in the HPLC analysis. Paclitaxel concentrations in these solutions were determined with the HPLC. Oils having higher solubility of paclitaxel were chosen especially in the formulation of the microemulsions.

#### 4.2.2. Preparation of Microemulsions

Different O/W nonionic microemulsions were formulated with surfactant mixtures and preselected oils having considerable solubility of paclitaxel. Suitable combinations of surfactants were sought to have higher equilibrium solubilities of aforesaid oils and more stable formulation. The apparent solubilities of oils in aqueous surfactant solutions were measured priorly to give an estimate on a possible work window on the concentration ranges of oils to be employed later in the formulation of the microemulsions. In sealed culture tubes, oils in excess were mixed, by a vortex mixer or a sonicator, with aqueous solutions of pure surfactants and surfactant mixtures in presence/absence of glycerol used as a co-surfactant. These test tubes were agitated on the end-to-end rotary mixer for about one day and left still for another day at 37 °C. Aliquots drawn from these samples were centrifuged at 6000 rpm to ensure complete separation of any insoluble oils. Apparent oil solubilities in these surfactant solutions were subsequently obtained by the HPLC analysis on these supernatants. It is noted that mixed ratios of surfactants and oils reported in this work were all based by weight, not by mole.

Phase diagrams were established from selected surfactants and oils to give convenience in making microemulsions. Certain amounts of surfactants, glycerol, oils, and deionized water were added into glass test tubes, which were gently mixed on the end-to-end rotary mixer for 2 h and, then, left still to reach equilibrium at 37 °C for one week or longer. Phase behaviors of these emulsion systems were mainly determined visually. Though microemulsions are thermodynamically stable, it is still difficult to define a clear-cut index that can be used to evaluate emulsion stability. The visual inspection and emulsion size are the commonly accepted indices for emulsion stability.

Aggregate size in selected samples was measured with the dynamic light scattering (DLS) technique over a period of a week to ensure the physical stability of these samples. Chemical stability of these samples was ascertained by monitoring changes in oil contents of these samples by HPLC. Stable systems were identified as those free of any physical phase-change, such as color change, phase separation, flocculation, and/or precipitation. It is of note that only clear or translucent emulsions were subject to DLS size measurements on microemulsions.

The stable microemulsion system would exhibit as bluish or translucent. Therefore, the phase boundary marking the stable microemulsion system was drawn between two adjacent composition points representing the stable microemulsion and the unstable agglomeration/two-phase regions.

#### 4.2.3. Solubilization of Paclitaxel by Microemulsion

Solubilization of paclitaxel was performed by adding paclitaxel in excess into culture tubes containing microemulsions without paclitaxel. Paclitaxel and the aforementioned microemulsions were mixed vigorously on a vortex mixer or by sonication operated at 40 kHz and with 200 W dissipation power (Delta DC200H, Delta New Instrument Co. Ltd., Taipei, Taiwan) for 10 min and, subsequently, on an end-to-end rotary mixer for a period of up to a week in an incubator at 37 °C. Prior to HPLC analysis, aliquots periodically withdrawn from resultant paclitaxel-containing microemulsions were filtered through with 0.45 μm PTFE syringe filters to remove any chunks of undissolved paclitaxel. The filtrates were then injected into the HPLC for the measurement of paclitaxel contents. From our preliminary data, apparent solubilities of paclitaxel in these microemulsions did not vary significantly after the third day from the onset of paclitaxel solubilization process.

### 4.3. Instrumental Analysis

The main instruments used in this work include high-performance liquid chromatography (HPLC) and the particle size analyzer based on the dynamic light scattering (DLS). HPLC analysis was perform to measure the concentrations of paclitaxel solubilized by microemulsions. The detailed information on the HPLC analysis was given in our previous report [4]. The stability of the microemulsions was monitored mainly with the size measurements using the DLS technique. The DLS analysis on the size of the aggregates was performed at 37 °C on the Zetasizer Nano ZS (Malvern Instruments Ltd., Malvern, UK). According to the manufacturer, the Zetasizer Nano ZS can provide reliable size analysis from 0.6 nm to 6 μm.

### 4.4. Cytotoxicity Studies

The cytotoxicity of the microemulsion formulations was examined with the procedures and the protocols reported in our previous report [4]. As before, the NIH/3T3 fibroblast cells (ATCC^®^ CRL-1658™, Manassas, VA, USA) were utilized in this work for the cytotoxicity assessments of selected microemulsion formulations. However, more cells (5000 cells per well) were initially plated in each well of the 96-well plate and incubated for 24 h at 37 °C. Subsequently, the media was replaced either with fresh culture media or microemulsions diluted with culture media to predetermined concentrations of total surfactants at 50 ppm and 200 ppm. Cells were incubated for 24 and 48 h, respectively, after which cell viability was measured using the MTT assay described in our previous report [4]. Student’s *t*-test was performed on the cell viability data with *p* < 0.05 set for statistical significance.

## 5. Conclusions

Solubilizers for the model hydrophobic drug, paclitaxel, were successfully demonstrated using nonionic microemulsions at 37 °C prepared from mixed surfactants and proper oil phases in this work. In brief, the model hydrophobic drug can be initially dissolved in a suitable oil phase that is, subsequently, microemulsified with appropriate surfactants. Consequently, a considerable amount of the hydrophobic drug could be encapsulated in the microemulsion. Thus, the apparent aqueous solubility of the hydrophobic drug could be substantially enhanced.

In this work, an acceptable amount of paclitaxel, around 70–100 ppm, was encapsulated in the prepared microemulsions at 37 °C, accounting for a significant improvement in comparison with the low aqueous solubility of paclitaxel (<0.3 ppm). Apposite conditions to prepare the microemulsions at 37 °C were identified from the phase diagrams of the mixed nonionic surfactants with tributyrin or ethyl caproate as the oil phases, constructed through a series of phase behavior experiments. The particle sizes of the obtained microemulsions were found mostly in the range from ca. 60 nm to ca. 200 nm. The cellular viability of 3T3 cells was investigated to give the assessment of the cytotoxicity of the microemulsion formulations. Accordingly, the cell viability of these microemulsion formulations with the concentration of total surfactants equal to 50 ppm and 200 ppm were still above 55% after 24 h. Therefore, the preparation principles of the microemulsions underlined in this this work could be applicable to prepare the solubilizers of the hydrophobic drugs.

## Figures and Tables

**Table 1:** Solubility of paclitaxel (PTX) in various oils and fatty esters at 37 °C.

Oils/Fatty Esters	Molecular Weight (g/mol)	PTX Solubility (mg/g·oil)	Health Risks of Oils and Fatty Esters
Ethyl Myristate	256.42	0.74	Non-hazardous
Ethyl Laurate	228.37	1.08	Non-hazardous
Ethyl Caprate	200.32	2.11	Mild skin irritation (rabbit, 24 h)
Ethyl Caprylate	172.27	5.84	No skin irritation (human)
Ethyl Caproate	144.21	14.57	Moderate skin irritation (rabbit, 24 h)
Triolein	885.43	0.28	LD_50_ = 12,000 mg/kg (rat, oral)
Tricaprylin	470.68	2.09	LD_50_ = 33,300 mg/kg (rat, oral)
Tributyrin	302.36	26.92	LD_50_ = 3200 mg/kg (rat, oral)
Peanut oil	Mixtures	0.50	Non-hazardous, edible
Grapeseed oil	Mixtures	0.33	Non-hazardous, edible
Sunflower oil	Mixtures	0.36	Non-hazardous, edible
Soybean oil	Mixtures	0.43	Non-hazardous, edible

LD_50_: Median Lethal Dose.

**Table 2:** Average sizes of various microemulsion formulations. Each formulation contains 2 wt % surfactant, 1 wt % glycerol, and different amounts of tributyrin or ethyl caproate.

**(1) Mixed Surfactant Systems Consisting of Pannox 74 and Tween 80**			
**Mixed Surfactant (w/w)** $\left(\frac{Pannox\;74}{Pannox\;74+Tween\;80}\right)$	**Oil Content**	**Aggregate Size (nm)**	
		**After 1 Day**	**After 7 Days**
0.2	0.3 wt % tributyrin	81.4	258.0
0.4	0.3 wt % tributyrin	79.2	87.2
0.5	0.3 wt % tributyrin	109.0	104.0
0.6	0.3 wt % tributyrin	87.5	240.0
0.8	0.3 wt % tributyrin	119.0	261.0
0.2	0.7 wt % ethyl caproate	96.6	110.5
0.4	0.7 wt % ethyl caproate	66.8	88.8
0.5	0.7 wt % ethyl caproate	59.4	63.4
0.6	0.7 wt % ethyl caproate	106.9	116.8
**(2) Mixed Surfactant Systems Consisting of Pannox 74 and Cremophor EL**			
**Mixed Surfactant (w/w)** $\left(\frac{Pannox\;74}{Pannox\;74+Cremophor\;\mathrm{EL}}\right)$	**Oil Content**	**Aggregate Size (nm)**	
		**After 1 Day**	**After 7 Days**
0.4	0.3 wt % tributyrin	188.8	216.9
0.5	0.3 wt % tributyrin	170.5	206.4
0.6	0.3 wt % tributyrin	270.2	274.9
0.4	0.5 wt % tributyrin	185.6	19 6.3
0.5	0.5 wt % tributyrin	200.7	195.4
0.6	0.5 wt % tributyrin	192.5	207.1
0.2	0.7 wt % ethyl caproate	101.9	82.4
0.4	0.7 wt % ethyl caproate	93.4	114.1
0.5	0.7 wt % ethyl caproate	233.5	301.4

**Table 3:** Encapsulation of paclitaxel (PTX) in various microemulsion formulations at 37 °C. Each formulation contains 2 wt % surfactant, 1 wt % glycerol, and different amounts of tributyrin or ethyl caproate.

**(a) Mixed Surfactant Systems Consisting of PANNOX 74 and Tween 80**				
**Mixed Surfactant (w/w)** $\left(\frac{Pannox\;74}{Pannox\;74+Tween\;80}\right)$	**Oil Content**	**Estimated PTX (ppm)**	**Measured PTX (ppm)**	**Solubilization Efficiency (%)**
0.2	0.3 wt % Tributyrin	79.8	52	65.2
0.4	0.3 wt % Tributyrin	79.8	61	76.5
0.5	0.3 wt % Tributyrin	79.8	71.2	89.2
0.6	0.3 wt % Tributyrin	79.8	57.3	71.8
0.8	0.3 wt % Tributyrin	79.8	53.5	67.1
0.2	0.7 wt % Ethyl Caproate	101.7	60.4	59.4
0.4	0.7 wt % Ethyl Caproate	101.7	76.8	75.5
0.5	0.7 wt % Ethyl Caproate	101.7	91.2	89.7
0.6	0.7 wt % Ethyl Caproate	101.7	73.8	72.6
**(b) Mixed Surfactant Systems Consisting of Pannox 74 and Cremophor EL**				
**Mixed Surfactant (w/w)** $\left(\frac{Pannox\;74}{Pannox\;74+Cremophor\;\mathrm{EL}}\right)$	**Oil Content**	**Estimated PTX (ppm)**	**Measured PTX (ppm)**	**Solubilization Efficiency (%)**
0.5	0.3 wt % Tributyrin	79.8	60.5	75.8
0.5	0.4 wt % Tributyrin	106.4	91.8	86.3
0.4	0.5 wt % Tributyrin	133.0	70.9	53.3
0.5	0.5 wt % Tributyrin	133.0	88.7	66.7
0.6	0.5 wt % Tributyrin	133.0	66.9	50.3
0.2	0.7 wt % Ethyl Caproate	101.7	63.9	62.8
0.4	0.7 wt % Ethyl Caproate	101.7	72.9	71.7
0.5	0.7 wt % Ethyl Caproate	101.7	82.8	81.4
0.4	0.9 wt % Ethyl Caproate	130.8	76.7	58.6

**Table 4:** Composition of selected microemulsion formulations used in viability of fibroblast 3T3 cells in Figure 3.

Microemulsion	Oil Phase	Nonionic Surfactant	Co-Surfactant
Formulation 1	0.3 wt % Tributyrin	2 wt %, Pannox74/Tween 80 = 1 (w/w)	1 wt % Glycerol
Formulation 2	0.7 wt % Ethyl Caproate	2 wt %, Pannox74/Tween 80 = 1 (w/w)	1 wt % Glycerol
Formulation 3	0.3 wt % Tributyrin	2 wt %, Pannox74/Cremophor EL = 1 (w/w)	1 wt % Glycerol
Formulation 4	0.7 wt % Ethyl Caproate	2 wt %, Pannox74/Cremophor EL = 1 (w/w)	1 wt % Glycerol
